# Tibia Multiplanar Deformities and Growth Disturbance Following Expandable Endoprosthetic Distal Femur Replacement

**DOI:** 10.3390/jcm11226734

**Published:** 2022-11-14

**Authors:** Ahmad Shehadeh, Muhamad Al-Qawasmi, Omar Al Btoush, Zeinab Obeid

**Affiliations:** 1 Department of Orthopedics, King Hussein Cancer Center, Queen Rania St. 202, Amman 11941, Jordan; 2 Department of Diagnostic Radiology, King Hussein Cancer Center, Queen Rania St. 202, Amman 11941, Jordan; 3 Department of Surgery, King Hussein Cancer Center, Queen Rania St. 202, Amman 11941, Jordan

**Keywords:** tibia deformity, growth disturbance, expandable prosthesis, distal femur, limb salvage, tibia discrepancy, sarcoma

## Abstract

Background: Expandable distal femur endoprosthesis (EDFE) is commonly used to compensate for the loss of the distal femoral epiphyseal plate in skeletally immature children who have undergone surgical resection of bone malignancies. However, the effect of the passive tibial component of the EDFE on tibial growth has not been extensively studied in the literature. This study aims to delineate the type, frequency, and associated risk factors of multiplanar proximal tibial deformities in skeletally immature children following the use of the expandable distal femur endoprosthesis (EDFE). Moreover, we plan to detect how these deformities influence the long-term functionality of the endoprosthesis in defining the need for subsequent implant revision or further surgical management. Patients and Methods: A total of 20 patients aged (7–12) years underwent expandable distal femur replacement. Two types of implants were used: Juvenile Tumor System (JTS) non-invasive prosthesis in 14 patients, and Modular Universal Tumor and Revision System (MUTARS)^®^ Xpand Growing Prostheses in six patients. A scanogram and CT scan documented the measurements of longitudinal and multiplanar growth as leg length discrepancy (LLD), femur length discrepancy (FLD), tibia length discrepancy (TLD), and the yield values of rotational, sagittal, and coronal deformities of the tibia. The patients were followed up to assess the need for further management. Sex, age, size of tibial plate perforation, and type of implant used were studied for possible correlation with deformities or growth disturbance. Results: The patients were followed up for a mean of 3 (2–7) years. A total of 14 patients, (10 JTS, 4 implant cast) had a tibial deformity and/or growth disturbance. A single patient was found to have all deformities (growth, rotational, coronal, and sagittal). Fourteen patients were found to have an LLD ranging from 5.3 to 59 mm (median 21 mm), 12 had a TLD from 3 to 30 mm, (median 10 mm), and 11 patients showed evidence of malrotation from 6 to 32 degrees (median 11 degrees). TLD was found to contribute entirely to LLD in three patients, and >50% of LLDs in seven patients. All LLDs were treated conservatively, except in three patients; two received contralateral tibia epiphysiodesis and one received revision with a new implant. A single patient had a posterior tibia slope angle (PTSA) of −2.8 degrees, and three patients had a coronal deformity with a mean medial proximal tibia angle (MPTA) of 80.3 (77–83 degrees). Conclusions: Tibial growth disturbance and multiplanar deformities occur in the majority of patients following EDFE replacement, exacerbating LLD. Yet, these disturbances may be well tolerated, managed conservatively, and rarely mandate endoprosthetic revision or subsequent corrective surgery. Age at the time of surgery was found to be the only significant contributor to the development of tibia growth disturbance.

## 1. Introduction

Limb salvage surgery tailored for skeletally immature patients with malignant tumors of the long bones presents a challenge in maintaining equivalent limb lengths when the growth period has ceased [1]. There are few surgical procedures allowing the preservation of growth plates, including osteogenesis, vascularized physeal transfer, and the use of expandable endoprosthetic implants, whereas amputation and rotationplasty remain the last choice when growth plates are unsalvageable in the case of advanced tumor stages [2].

In children, the distal femur remains the most common site for malignant bone tumors, namely, osteosarcoma. Limb salvage surgery with reconstruction stands as the gold standard, especially in children who have reached full growth potential. However, in patients with an active growth plate, tumor excision within the area may disrupt limb length, resulting in functional deficit and gait disturbances [3].

Disruption of the growth plate by tumor invasion and therapeutic limb salvage techniques affects normal longitudinal physeal growth, which averages 13 mm per year in the distal femur and 9 mm per year in the proximal tibia [4]. Surgical techniques utilize expandable distal femur endoprosthesis to account for the halted growth of the distal femur. EDFE has been evolved to provide an elongation of the limb that does not necessitate subsequent surgery or anesthetics.

EDFE requires stemming of the tibia proximal growth plate through smooth metallic surfaces such as those of XPAND, MUTARS, or sliding stems inside a polyethylene tube that penetrate the proximal tibia growth plate, as that of JTS. In addition to the longitudinal growth disturbances of the femur, discrepancies in length and angulation may occur in the proximal tibia too. Arteau et al. confirm the halt in the growth of the proximal tibial physis upon insertion of the endoprosthetic stem through the physis [2].

Therapeutic measures to combat growth disturbances do not commonly necessitate surgical revision. In clinical practice, orthotic devices or epiphysiodesis of the contralateral limb may suffice [5].

The purpose of this study is to assess the longitudinal and multiplanar tibial growth disturbance following distal femur excision and reconstruction using expandable distal femur endoprosthesis. Therefore, we evaluated the occurrence, number, and type of the tibia multi-planar growth disturbance and noted the correlation between patient demographics, the size of tibia epiphysis perforation, and implant type. Moreover, we assessed the contribution of tibial length discrepancy (TLD) to the total leg length discrepancy (LLD) and mapped out possible therapeutic modalities.

## 2. Materials and Methods

This is a retrospective cohort study that utilized the pre-existing medical records of 20 skeletally immature patients who have undergone distal femur replacement using an expandable implant at the King Hussein Cancer Center between 2009 and 2019.

Nonsurgical treatment included neoadjuvant chemotherapy. MAP and VDC/IE protocols were applied for osteosarcoma and Ewing sarcoma accordingly. HDMTX-CF, adriamycin (ADM)+ cisplatin (DDP), and ifosfamide (IFO) were prescribed for osteosarcoma and AVI ((ADM, VP-1, IFO) for Ewing’s sarcoma [6]. The operation was performed later, after the chemotherapy for the patient’s recovery. All patients received neoadjuvant chemotherapy per KHCC protocol.

The patients’ demographic data and clinical features were obtained from medical records. This also included information on treatment regimens and post-treatment occurrences with the approval of The Institutional Review Board (IRB) at the King Hussein Cancer Center (KHCC-IRB), proposal No.21 KHCC 105, 11 July 2021. Limb length discrepancy (LLD), tibial length discrepancy (TLD), coronal deformities, sagittal deformities, and rotational deformities were investigated. A computed topography (CT) scan was utilized to measure the preoperative diameter of the proximal tibial epiphyseal perforation corresponding to the size of the implant stem. 

All patients underwent the objective tools of scanogram imaging and computed tomography (CT) scans of both legs to assess longitudinal disturbances and multiplanar deformities; this is to ensure accurate assessment and appropriate alignment after surgery [7]. Several recent papers have used the supine CT scanogram to assess lower limb alignment, which has proved to have higher levels of accuracy compared to other methods. Accuracy is crucial for having a good lengthening procedure during follow-ups [8,9,10]. The growth disturbance indices are defined as:

1. *Leg length discrepancy (LLD)* is defined as a difference of 3 mm or more and is measured from the center of the femur head to the center of the ankle. This was calculated by subtracting the length of the operative limb from the length of the contralateral limb.
2. *Femur length discrepancy (FLD)* is calculated by subtracting the length of the operative femur from the length of the contralateral femur. A negative value was given when the operative femur was longer than the opposite side.
3. *Tibial length discrepancy (TLD)* is defined as a difference in the tibial length of 3 mm or more. A discrepancy of less than 3 mm was attributed to physiological variations or errors in measurement. The tibial length was calculated from the middle of the proximal tibia epiphysis to the center of the distal tibia epiphysis.
4. *Coronal deformity (Tibia varus deformity)* is defined as a medial proximal tibial angle (MPTA) of less than 85 degrees. The angle is measured between the anatomical axis of the tibia and the tangential axis of the tibia epiphysis.
5. *Sagittal deformities are defined* as a posterior tibial slope angle (PTSA) less than 0 (normal range 0–14 degrees). The angle is measured between the anatomical axis of the tibia on the lateral view and the tangential line from the anterior to posterior of the tibia plateau.
6. *The rotational deformity* is defined as a difference of more than five degrees in the rotational angle between the operative side and the contralateral leg. The angle is present between the long axis of the proximal tibia metaphysis cross-section and the horizontal line.

### 2.1. Endoprosthesis

**Figure 1:**
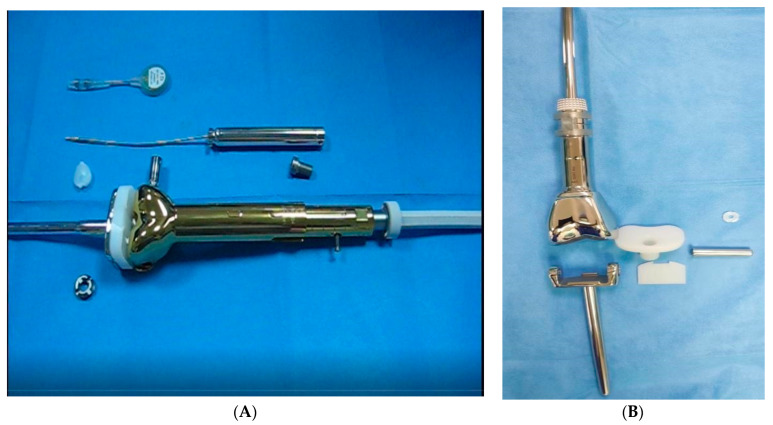
(**A**): The MUTARS^®^ Xpand Growing Prostheses (on the right). (**B**): The Juvenile Tumor System (JTS) (on the left).

Two types of endoprosthesis were used: Modular Universal Tumor and Revision System (MUTARS)^®^ Xpand Growing Prostheses by ImplantCast and Juvenile Tumor System (JTS) non-invasive prosthesis by Stanmore. Both implant mechanisms use a femoral and tibial component simulating a total knee replacement. The MUTARS^®^ Xpand Growing Prostheses employ a hinged-knee mechanism consisting of a polyethylene sleeve overlying a stemmed metal tibial baseplate (Figure 1A). This implant was only used in six out of 20 patients. The remaining 14 patients received the Juvenile Tumor System (JTS), a product of Stanmore in which the tibial component is a sliding metallic stem inside a polyethylene tibial baseplate tube with rotational capabilities (Figure 1B). According to previous studies, the mean achieved elongation of the limb was found to be 36.4 mm in MUTARS and 25 mm in JTS [11,12].

### 2.2. Surgical Details

All patients underwent limb salvage surgery coupled with EDFE, as performed by a single surgeon at a single institution (our senior author (AS)). The type, size, and utilization of EDFE for each patient are based on preoperative imaging, anticipated resection, and the possibility of leg length discrepancy. Complete resection of the distal femur was achieved in adherence with our own previously published surgical techniques [12,13,14]. None of the patients had preoperative mechanical or anatomical deformities. Therefore, in the preparation for the tibia, we resected the cartilaginous surface of the proximal tibial epiphysis, persevering a bulk of the epiphysis and epiphyseal plate. Additionally, we created a central entry point in the epiphysis and epiphyseal plate that ranged between 10.5 mm and 16.5 mm in diameter. All patients were rehabilitated postoperatively using our standardized rehabilitation protocol [14].

### 2.3. Statistical Analysis

A descriptive analysis of patients to include categorical data such as gender, prosthesis type, and other factors was presented as counts and percentages. The mean and range were calculated for the continuous data, including the age and diameter of tibial plateau entry.

Differences in proportions were tested with the χ2 test or Fisher exact test. Similarly, differences in continuous variables were tested with a Student’s *t*-test or nonparametric test (Wilcoxon Rank Test) depending on the assumption required for each test. A significant criterion of *p* ≤ 0.05 was used in the analysis. All analyses were performed using SAS version 9.4 (SAS Institute Inc., Cary, NC, USA).

## 3. Results

All patients received expandable endoprosthesis to minimize reconstructive challenges and complications. General associated complications are failure of expansion (15%), aseptic loosening of the femoral stem (15%), proximal tibial fracture (5%), stiffness of the knee joint (5%), recurrence (5%), and ultimately needed above-knee amputation. In this study, a total of 20 patients underwent expandable distal femur replacement for high-grade osteosarcoma (*n* = 19) and Ewing sarcoma (*n* = 1). The mean age at diagnosis was 9.65 ± 1.8 with a male-to-female ratio of 1:1. The mean follow-up period was 3 years, ranging from 2 to 7 years. R0 resection was achieved in all patients according to postoperative histopathology. Distant metastasis was found in three patients; two cases had pulmonary metastasis and one case had bone metastasis. One patient had local recurrence. Four patients died of the dissemination of tumors. Overall survival was 80%.

### 3.1. Presence, Frequency, and Risk Factors for Growth Disturbance or Deformities

**Figure 2:**
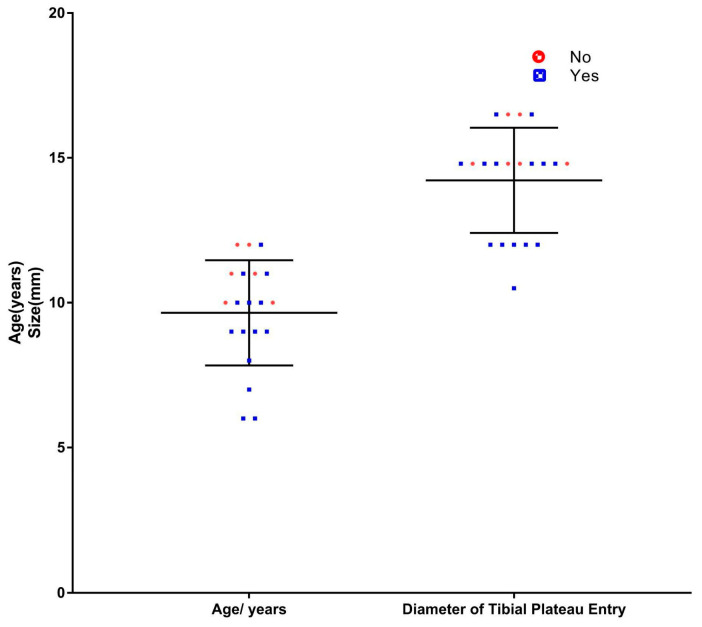
A scatter plot depicting the presence of an association between deformities and age but no significant association between deformities and tibial plateau entry diameter.

A total of 14 patients (70%) in our cohort had one or more growth disturbances or deformities (Table 1). Patients who were ages 9.1 years and younger at the time of surgery had an increased incidence of deformities and growth disturbance (*p* = 0.019) (Figure 2). No significant difference was noted in the presence of deformities between male patients and female patients (8; 57.1% vs. 6; 42.9%) (*p* = 0.999). Moreover, the mean resection length for the 14 patients who had tibial growth distarbance was 173 mm (160–185 mm), while the mean resection length for the 6 patients who did not have a growth disturbance was 162 mm (137–168 mm). However, the resection length was not found to be statistically a contributing factor for the occurrence of the tibial growth disturbance (*p* = 0.465). The mean diameter of tibial plateau entry for patients who had the deformities was 13.7 mm (12.8–14.4 mm). Whilst for the patients who did not have the deformities, the mean was 15.4mm (14.6–16.2 mm), which is statistically insignificant (*p* = 0.074). (Table 2).

### 3.2. Leg Length Discrepancy (LLD)

Leg length discrepancy was noted in 13 patients, with an average of 28 mm ranging from 5.3 to 59 mm. These values were greater than 30 mm in four of the patients. The cause of LLD > 30 mm was identified in three patients as a failure of the expansion mechanism yielding an inability to elongate the operative limb. Two of these patients underwent or were planned to undergo revision. The last patient was treated with epiphysiodesis of the contralateral distal femur and proximal tibia to stop the increase in the discrepancy.

### 3.3. Femur Length Discrepancy (FLD)

Femur length discrepancy was found in 13 patients, (−10 mm to 49 mm). In two patients, the discrepancy was due to excessive elongation of the femur to compensate for the shortening of the ipsilateral tibia (patient 2, 6). Six patients had an FLD of more than 10 mm, three due to failure of the expansion mechanism (patients 1, 8, 10), and the remaining three due to reaching the maximum stroke (patients 9, 12, 13).

### 3.4. Tibial Length Discrepancy (TLD)

**Figure 3:**
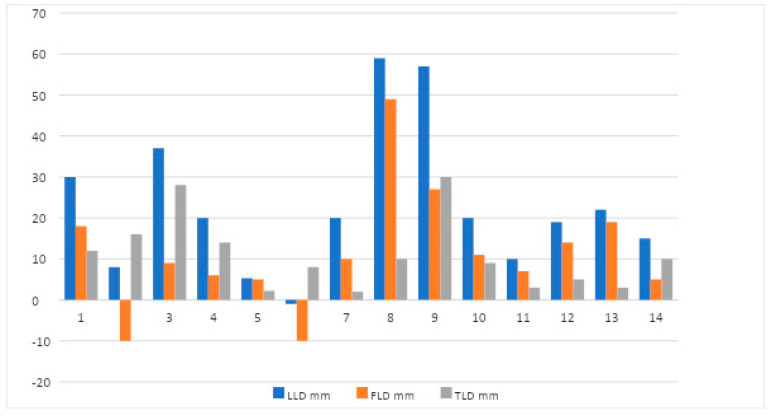
A histogram of the 14 patients who showed radiological evidence of leg length discrepancy.

**Figure 4:**
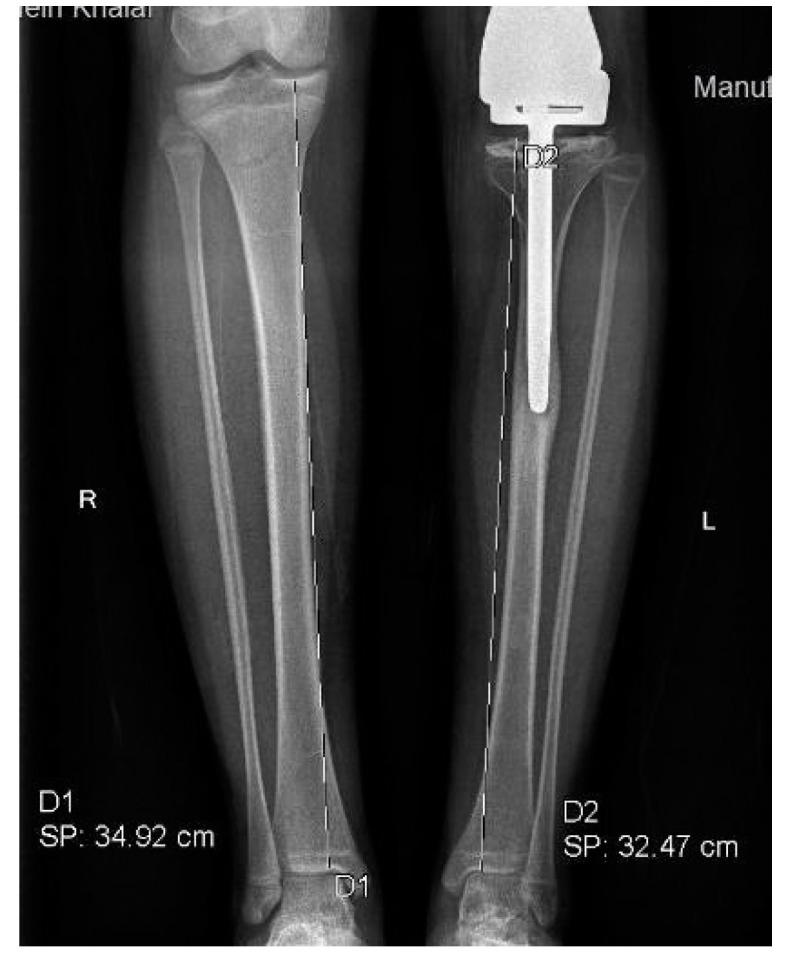
Tibial length discrepancy of 24.5 mm in one of our patients.

Tibial length discrepancy was discovered in 13 patients with a mean of 12 mm and a median of 10 mm (3–30 mm) (Figure 3). Only three patients had a TLD of 20 mm or greater, three had a TLD of more than 10 mm and less than 20 mm, and the remaining patients showed values of <10 mm. Twelve out of the 13 patients with LLD had concurrent TLD, contributing to 13–100% (59%) of the difference in length. In three patients, LLD was caused entirely by TLD. A histogram of the magnitude of growth disturbance indices in 14 patients is depicted in Figure 4.

### 3.5. Rotational and Angular Deformities

**Figure 5:**
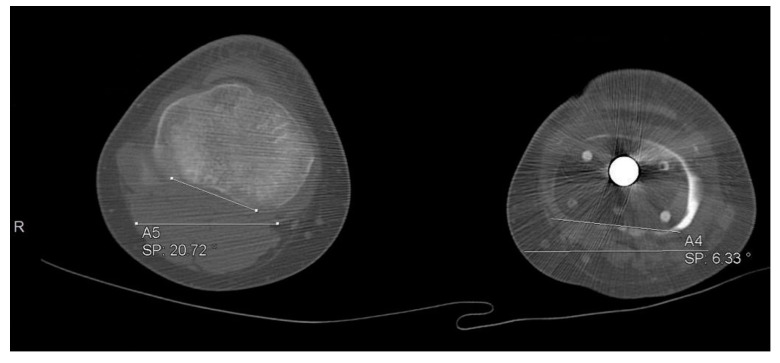
Rotational deformity of 26 degrees in one of our patients.

**Figure 6:**
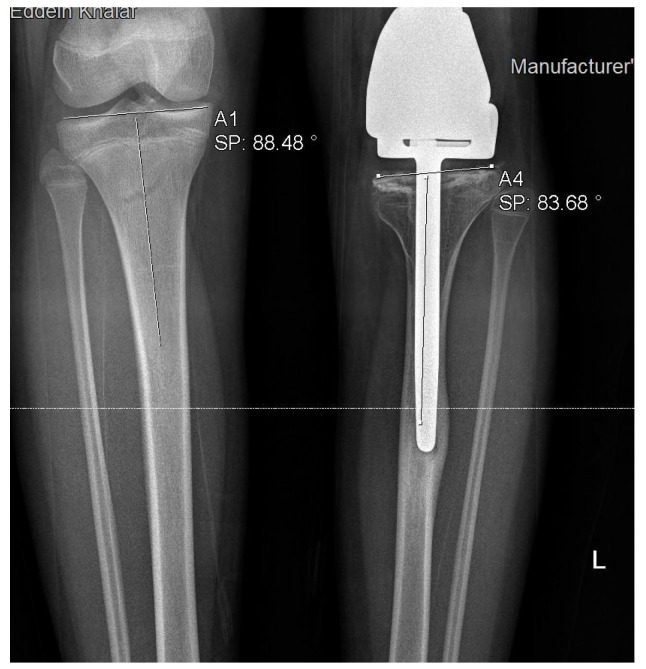
Coronal varus deformity in one of our patients.

Eleven patients had tibia rotational deformities with a mean difference of 13.4 degrees (632 degrees) between the operative and contralateral sides (Figure 5). Coronal deformities were detected in three patients, with a mean MPTA of 80.3 degrees (77–83 degrees) (Figure 6). One patient had a sagittal deformity of PTSA six degrees.

### 3.6. Need for Further Management

**Figure 7:**
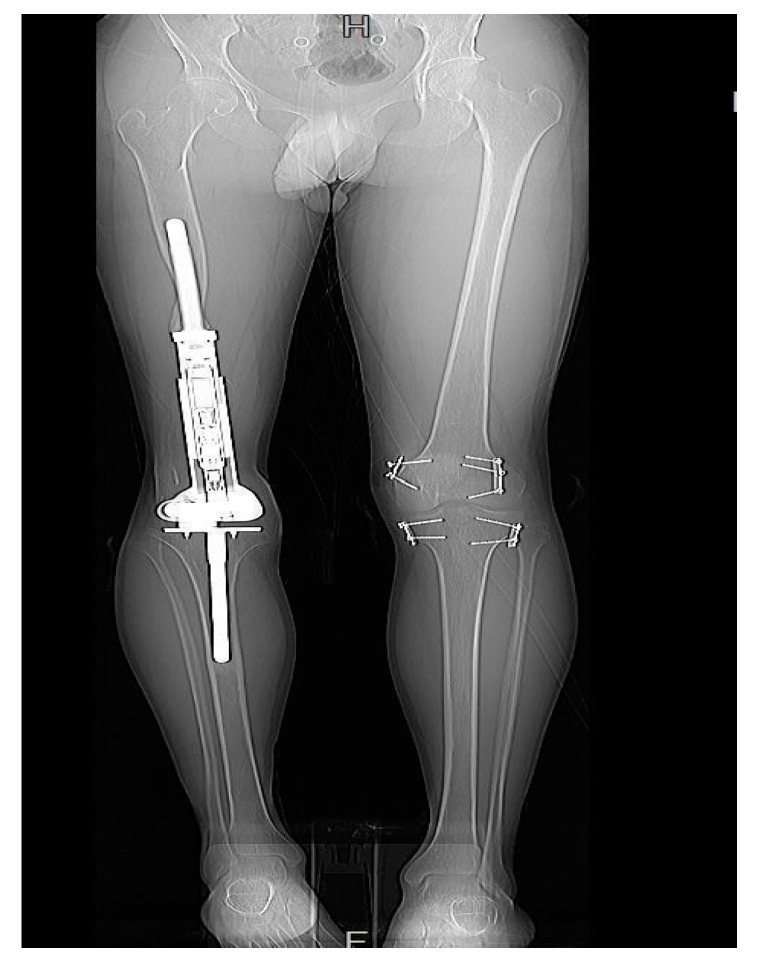
Patient 3 had undergone EDFE using MUTARS^®^ Xpand. However, due to an LLD of 37 mm, this patient required epiphysiodesis of the contralateral limb.

Patients with rotational and angular deformities did not necessitate management, as the deformities were tolerated well. Moreover, patients with an LLD of less than 10 mm demanded no further management. An LLD between 10 and 20 mm was managed solely by a shoe lift. However, an LLD of >20 mm, was treated on a case-by-case basis. Two patients required operative revision and serial elongation due to the anticipated growth potential of the contralateral leg. The remaining two patients were managed using epiphysiodesis of the contralateral distal femur and proximal tibia (Figure 7).

## 4. Discussion

Evidence on the occurrence of tibial growth disturbances and deformities following the use of expandable distal femur implants is scarce. To the best of our knowledge, this is the first article encompassing multiplanar tibial deformities in skeletally immature patients. We analyzed the possible factors correlated with the incidence and magnitude of these deformities and the postoperative therapeutic modalities needed. 

### 4.1. Presence, Frequency, and Risk Factors for Growth Disturbance or Deformities

We found the prevalence of deformities in over two-thirds of patients who received EDFE replacement with a predominance of combined growth abnormalities. TLD occurred in 93% of all patients with LLD contributing to more than 50% of the length discrepancy in half of the patients.

In almost one-fourth of our patients, TLD was the only cause of LLD. We found an average LLD of 28 mm in contrast to Ji et al., with about 53 mm following the use of non-hinged endoprosthesis [5].

Patient age that was less than or equal to 9 years was the primary factor associated with a disruption of leg length indices. We attribute the effect of age to skeletal maturity, as reduced growth plate potential minimizes the risk of deformities [1]. Sex, implant type, entry size and resection length were not shown to be substantially associated with the incidence of these abnormalities. Although radiological evidence proves the presence of a dense cortical reaction upon tibial stemming, our findings did not report a significant association between the tibial plateau entry size, resection length, and the presence of deformities [17,18]. The current literature on expandable endoprostheses infrequently mentions secondary angular deformity [2,3,11,19,20,21,22,23,24,25,26]. Arteau et al.’s study showed that angular deformity may occur in both the sagittal and coronal planes. The pathogenesis may be related to asymmetric physeal growth and small physeal bars that are hidden by radiopaque implants. Once angular deformity occurs, asymmetric compressive forces may cause further growth inhibition, according to the Heuter-Volkmann principle [2].

### 4.2. Need for Further Management

None of the patients with angular or rotational deformities required additional surgery. Although the impact of limb length discrepancy and angular deformity on long-term functional outcomes and health-related quality of life is uncertain, they are still worth noting. 

**Figure 8:**
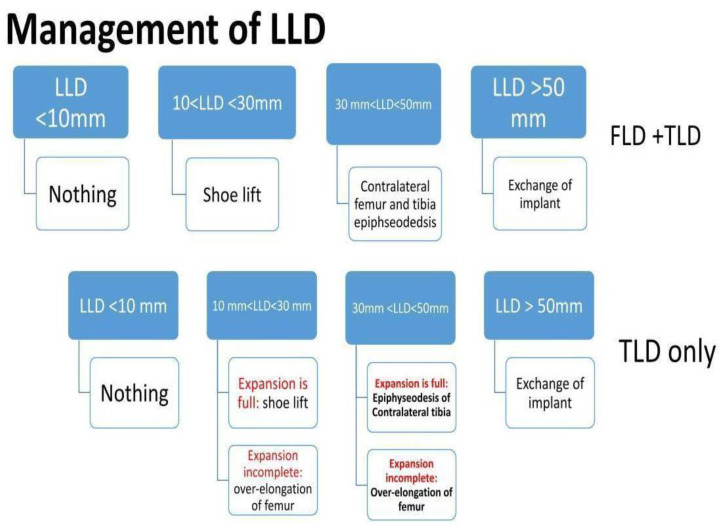
Management of LLD.

A TLD of greater than 20 mm may be effectively managed by over-elongation of the expandable femur, provided adequate stroke length remains. However, in case that the expansion mechanism has reached its full capacity, epiphysiodesis may be employed. Epiphysiodesis is used to account for the residual discrepancy in patients with remaining growth potential with an efficacy of 66% at maturity in both the femur and tibia [27]. This therapeutic modality bears a complication rate of 7%, commonly resulting in the development of angular deformities [28]. Additionally, we reserve shoe lifts only when the family is opposed to epiphysiodesis, although this method is largely used in an LLD of <20 mm [29]. However, studies in skeletally mature patients have yielded no significant functional effect of this intervention [30]. A TLD of less than 20 mm is often tolerated by the patient’s compensatory mechanisms. If the total LLD was greater than 30 mm, epiphysiodesis of the contralateral distal femur alone or in conjunction with the contralateral proximal tibia might be warranted. We have utilized this treatment option in two of our patients and appreciated satisfactory results. Further elongation of the surgical femur was performed in three patients to correct operative limb shortening caused by TLD (Figure 8).

In only one patient in our series, complete revision with a new expandable implant was carried out to compensate for a length discrepancy of 50 mm between the lower limbs. This was in contrast to the findings in the literature where 23.1% of patients required surgical revision [31]. Gupta et al. deemed revision unnecessary for JTS implants in cases where the patient growth potential exceeded the implant’s limit [11,18].

Our study had several limitations, including the retrospective nature of the study and a relatively small number of patients, so the risk of bias is high and statistical analyses are limited. Four of our patients died within the follow-up period. The determination of deformity growth may differ between scanograms, leading to inaccurately measuring tibial length. In addition, it is a single-institutional study with a short follow-up period. Moreover, the need for further observation is required to determine whether a patient will be able to function with a pediatric size endoprosthesis in the long-term.

## 5. Conclusions

In conclusion, tibial growth disturbance and the occurrence of angular and rotational deformities are frequent upon expandable distal femur replacement. The contribution of inadequate tibial growth on LLD is demonstrated in the majority of patients and should be accounted for during surgical planning. Therefore, it is possible to predict the need for increased expansion of the implant to accommodate the anticipated growth deficiency of the ipsilateral tibia.

We found that the majority of growth abnormalities may be managed non-surgically, as they are tolerated well by the patient. Multiplanar deformities are common, but they may not be clinically significant. Statistical significance does not denote clinical significance [7]. Thus, the bulk of cases will seldom necessitate subsequent surgical correction. Effective non-surgical treatment options include excessive elongation of the expandable femur and shoe lift.

Larger multi-center studies are warranted to identify the magnitude of risk factors for the occurrence of these deformities and delineate preventative measures to minimize their occurrence.

In general, surgical corrective procedures including revision or epiphysiodesis were used in a minority of patients.

## Figures and Tables

**Table 1:** Patients’ leg length discrepancy data and surgical details [15,16].

Patient Number	No. of Deformities	Sex/Age	Implant	Resection Length mm	Diameter of Tibial Plateau Entry	LLD mm	FLD mm	TLD mm	TLD as Percentage of Total LLD	Status of Device
1	3	M/11	JTS	170	14.8	30	18	12	40%	Failed
2	2	F/11	MUTARS	190	12	8	−10	16	100%	Completed
3	3	M/10	MUTARS	150	12	37	9	28	75%	Failed
4	4	M/9	JTS	230	14.8	20	6	14	70%	Completed
5	2	F/9	JTS	160	14.8	5.3	5	2.2		Ongoing
6	2	M/12	JTS	210	16.5	−1	−10	8	100%	Completed
7	3	M/6	JTS	150	10.5	20	0	20	100%	Completed
8	3	F/9	JTS	170	14.8	59	49	10	17%	Failed
9	3	F/6	JTS	160	14.8	57	27	30	52%	Completed/exchanged
10	3	M/7	JTS	150	12	20	11	9	45%	Failed
11	2	M/10	JTS	175	16.5	10	7	3	30%	Ongoing
12	2	F/10	MUTARS	200	12	19	14	5	26%	Completed
13	2	F/9	JTS	170	14.8	22	19	3	13%	Completed
14	1	M/8	MUTARS	130	12	15	5	10	67%	Completed

**Table 2:** Association between the presence of deformities and patient characteristics [15,16].

		Presence of Deformities		*p*-Value
		No	Yes	
Gender	F	3 (50.0%)	6 (42.9%)	0.999
	M	3 (50.0%)	8 (57.1%)	
Prosthesis type	JTS	4 (66.7%)	10 (71.4%)	0.999
	MUTARS^®^ Xpand	2 (33.3%)	4 (28.6%)	
Age/years	Mean (95% CI)	11.0 (10.3, 11.7)	9.1 (8.2, 9.9)	0.019
Diameter of Tibial Plateau Entry	Mean (95% CI)	15.4 (14.6, 16.1)	13.7 (12.8, 14.6)	0.074
Average of resection length	Mean (172 mm)	162 (137, 186)	173 (160, 185)	0.465
Average expansion length	Mean 50 mm	-	-	NA

## Data Availability

The datasets generated and used for the current study are available from the corresponding author upon request.
